# Disulfide bond-disrupting agents activate the tumor necrosis family-related apoptosis-inducing ligand/death receptor 5 pathway

**DOI:** 10.1038/s41420-019-0228-9

**Published:** 2019-12-10

**Authors:** Mengxiong Wang, Mary E. Law, Bradley J. Davis, Elham Yaaghubi, Amanda F. Ghilardi, Renan B. Ferreira, Chi-Wu Chiang, Olga A. Guryanova, Daniel Kopinke, Coy D. Heldermon, Ronald K. Castellano, Brian K. Law

**Affiliations:** 1 Department of Pharmacology, University of Florida, Gainesville, FL USA; 2 Department of Chemistry, University of Florida, Gainesville, FL USA; 3 Institute of Molecular Medicine, College of Medicine, and Center for Infectious Disease and Signaling Research, National Cheng Kung University, Tainan, Taiwan; 4 UF-Health Cancer Center, University of Florida, Gainesville, FL USA; 5 Department of Medicine, University of Florida, Gainesville, FL USA

**Keywords:** Breast cancer, Oncogenes

## Abstract

Disulfide bond-disrupting agents (DDAs) are a new chemical class of agents recently shown to have activity against breast tumors in animal models. Blockade of tumor growth is associated with downregulation of EGFR, HER2, and HER3 and reduced Akt phosphorylation, as well as the induction of endoplasmic reticulum stress. However, it is not known how DDAs trigger cancer cell death without affecting nontransformed cells. As demonstrated here, DDAs are the first compounds identified that upregulate the TRAIL receptor DR5 through transcriptional and post-transcriptional mechanisms to activate the extrinsic cell death pathway. At the protein level, DDAs alter DR5 disulfide bonding to increase steady-state DR5 levels and oligomerization, leading to downstream caspase 8 and 3 activation. DDAs and TRAIL synergize to kill cancer cells and are cytotoxic to HER2+ cancer cells with acquired resistance to the EGFR/HER2 tyrosine kinase inhibitor Lapatinib. Investigation of the mechanisms responsible for DDA selectivity for cancer cells reveals that DDA-induced upregulation of DR5 is enhanced in the context of EGFR overexpression. DDA-induced cytotoxicity is strongly amplified by MYC overexpression. This is consistent with the known potentiation of TRAIL-mediated cell death by MYC. Together, the results demonstrate selective DDA lethality against oncogene-transformed cells, DDA-mediated DR5 upregulation, and protein stabilization, and that DDAs have activity against drug-resistant cancer cells. Our results indicate that DDAs are unique in causing DR5 accumulation and oligomerization and inducing downstream caspase activation and cancer cell death through mechanisms involving altered DR5 disulfide bonding. DDAs thus represent a new therapeutic approach to cancer therapy.

## Introduction

Drugs exist for tumors overexpressing human epidermal growth factor receptor 2 (HER2) or epidermal growth factor receptor (EGFR) tyrosine kinases. However, drug resistance is a common problem. Tumors that lack estrogen receptor, progesterone receptor, and HER2 are termed “Triple-Negative” Breast Cancers (TNBCs). TNBCs resist most targeted therapies. EGFR is overexpressed in a substantial fraction of TNBCs and is a potential therapeutic target^1^, but EGFR-targeted therapies have not exhibited sufficient activity against EGFR+ TNBCs. EGFR and HER2-specific drugs act through the mechanism of oncogene addiction. Cancers often escape from oncogene addiction. Synthetic lethality relies on drug actions that manifest selectively in the context of tumor-associated genetic alterations. Given the current limitations of EGFR and HER2-targeted drugs in treating breast cancer, new agents that act through a synthetic lethal mechanism could benefit patients with EGFR+ TNBCs and drug- resistant HER2+ tumors.

Previous reports^2–4^ describe a novel class of anticancer agents termed disulfide bond-disrupting agents (DDAs). DDAs kill cancer cells that overexpress EGFR or HER2^4^, and DDAs decrease expression of EGFR, HER2, and HER3 and reduce phosphorylation of the pro-survival kinase Akt. DDA-mediated cancer cell death is also associated with activation of the unfolded protein response (UPR)^2^. The endoplasmic reticulum (ER) stress that triggers the UPR can activate multiple cell death pathways (reviewed in ref. ^5^). However, the specific cell death mechanisms engaged by the DDAs remain unexplored.

Irremediable ER stress can drive apoptosis by upregulating the death receptor 5 (DR5) protein through transcriptional mechanisms^6–9^. Activation of DR5 by its ligand, TNF-related apoptosis-inducing ligand (TRAIL), selectively kills cancer cells without effects on nontransformed cells, and shows manageable side effects in clinical trials^10–12^. However, TRAIL and other DR5 agonists have not met expectations in clinical trials, in part because cancer cells can easily become TRAIL resistant by downregulating DR5^13–15^. A strategy for increasing DR5 levels and activating downstream DR5 apoptotic signaling could bypass the resistance to TRAIL and DR5 agonist antibodies observed in the clinic.

## Results

### DDAs activate the extrinsic apoptosis pathway to kill EGFR+ and HER2+ cancers

**Fig. 1:**
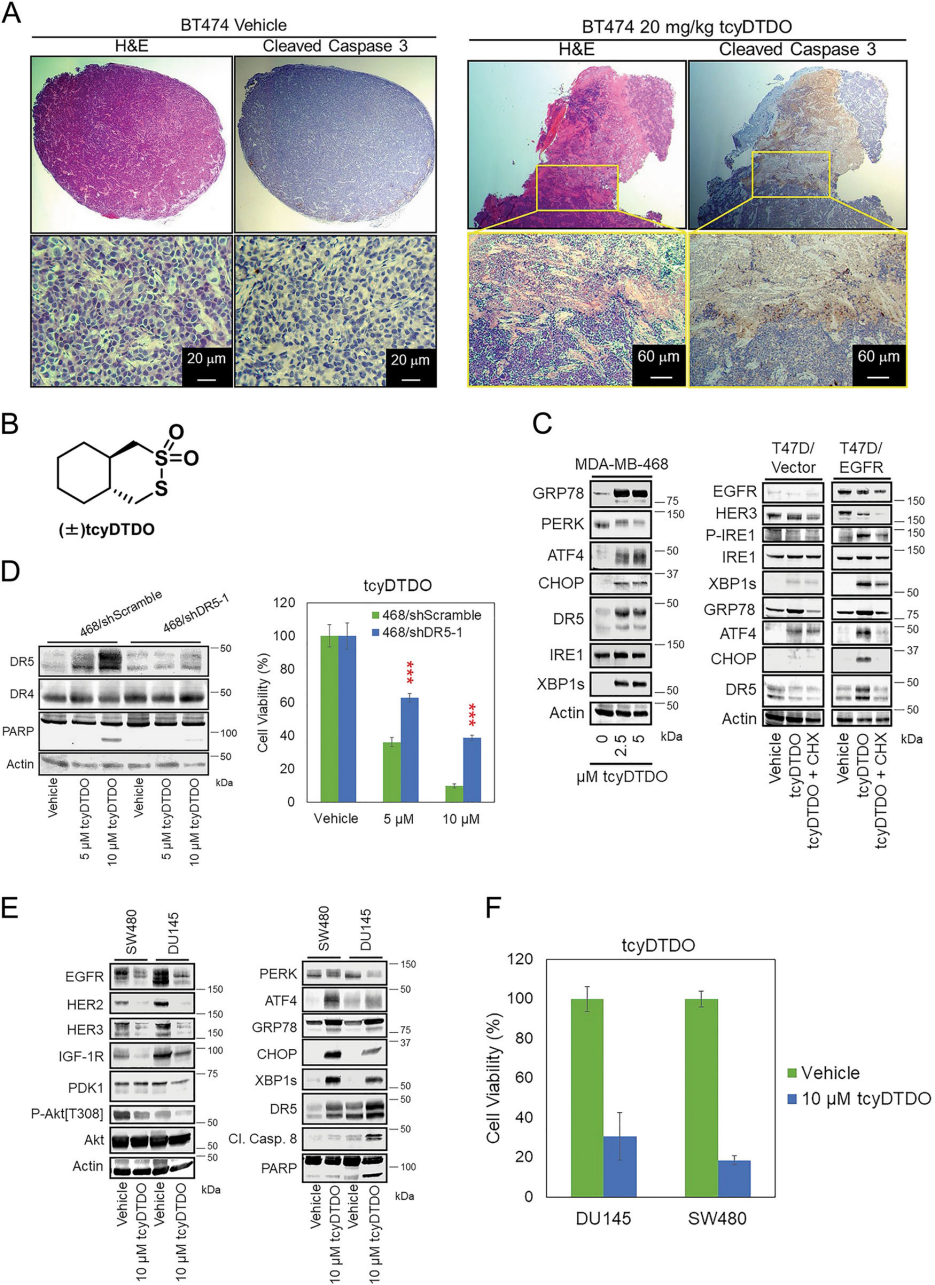
DDAs activate the extrinsic apoptosis pathway to kill EGFR+ and HER2+ cancers. **a** Tumor-bearing mice were randomly separated into two groups of three mice each and treated once daily for 5 days with either vehicle (DMSO) or 20 mg/kg tcyDTDO. Mice were killed on day 5 (3 h after treatments) and tumor samples were collected for hematoxylin and eosin (H&E) staining and cleaved caspase 3 staining. **b** Structure of DDA tcyDTDO. **c** MDA-MB-468 and the indicated T47D stable cell lines treated for 24 h have been indicated and cell extracts were analyzed by immunoblot. **d** Left panel. Immunoblot analyses were performed on the indicated MDA-MB-468 lentivirally transduced shRNA knockdown cell lines with 5 or 10 µM tcyDTDO treatments. Right panel. MTT assays were performed on the same cell lines treated with the indicated concentrations of tcyDTDO for 72 h. **e** SW480 and DU145 cell lines were treated as indicated for 24 h and analyzed by immunoblot. **f** MTT assays were performed on SW480 and DU145 cells with indicated treatments for 72 h. The results are presented as the mean ± standard deviation of five replicate determinations. Significance was determined using Student’s *t* test with ***P ≤ 0.001.

Breast cancer cells that overexpress EGFR or HER2 are sensitive to DDAs^2,4,16^. Past work employed MDA-MB-468 TNBC cells and BT474 luminal B cells as models of EGFR and HER2-overexpressing breast cancer, respectively. Consistent with these previous studies, DDAs show extensive anticancer effects in vivo. Mice bearing orthotopic xenograft BT474 tumors were treated with vehicle (DMSO) or different doses of our most highly potent DDA tcyDTDO^16^ for 5 days. Hematoxylin and eosin (H&E) histochemical staining revealed that the tumors were substantially necrotic after tcyDTDO treatment (Fig. 1a, left panels). Cleaved caspase 3 staining indicated tumor cell death due to apoptosis (Fig. 1a, right panels). To investigate which pathway mediates DDA-induced tumor cell death, we screened multiple cell death axes. Immunoblot analyses showed that tcyDTDO (Fig. 1b) significantly increased the levels of DR5 (Fig. 1c, left panel), suggesting that DDAs activate extrinsic apoptosis in cancer cells. T47D cells model low EGFR/HER2 luminal A cancers^2,4,16^. These studies showed that T47D cells overexpressing EGFR or HER2 are highly sensitive to DDAs. Therefore, we examined whether DR5 expression increases in EGFR-overexpressing T47D cells treated with DDAs. TcyDTDO activated ER stress and upregulated DR5 in T47D/EGFR cancer cells, but not T47D/vector cells (Fig. 1c, right panel). The combination of tcyDTDO and Cycloheximide (CHX), a protein synthesis inhibitor, reversed DDA-induced upregulation of ER stress and DR5, indicating that DDA-triggered ER stress is responsible for increasing DR5 levels. To confirm that DR5 is critical for regulating the sensitivity of cancer cells to DDAs, we generated DR5 knockdown MDA-MB-468 lines (Fig. 1d). Knockdown of DR5 decreased PARP cleavage and partially reduced sensitivity of cancer cells to tcyDTDO treatments in MTT assays (Fig. 1d).

We previously demonstrated that DDA treatment kills cancer cells that overexpress EGFR and/or HER2^16^. The human tumor lines DU145 (prostate) and SW480 (colon) are DDA-sensitive non-breast cancer cell lines. TcyDTDO downregulated the expression levels of EGFR/HER2/HER3, induced Akt dephosphorylation, and caused ER stress in these cells (Fig. 1e). Importantly, tcyDTDO also triggered the extrinsic apoptosis pathway as measured by the upregulation of DR5, CC8, and PARP cleavage, and significant reduction of cell viability (Fig. 1f). These data suggest that DDAs activate the extrinsic apoptosis pathway to kill EGFR+ and HER2+ cancers.

### DDAs upregulate DR5 through both transcriptional and post-transcriptional mechanisms, by altering DR5 disulfide-bonding pattern and promoting its oligomerization

**Fig. 2:**
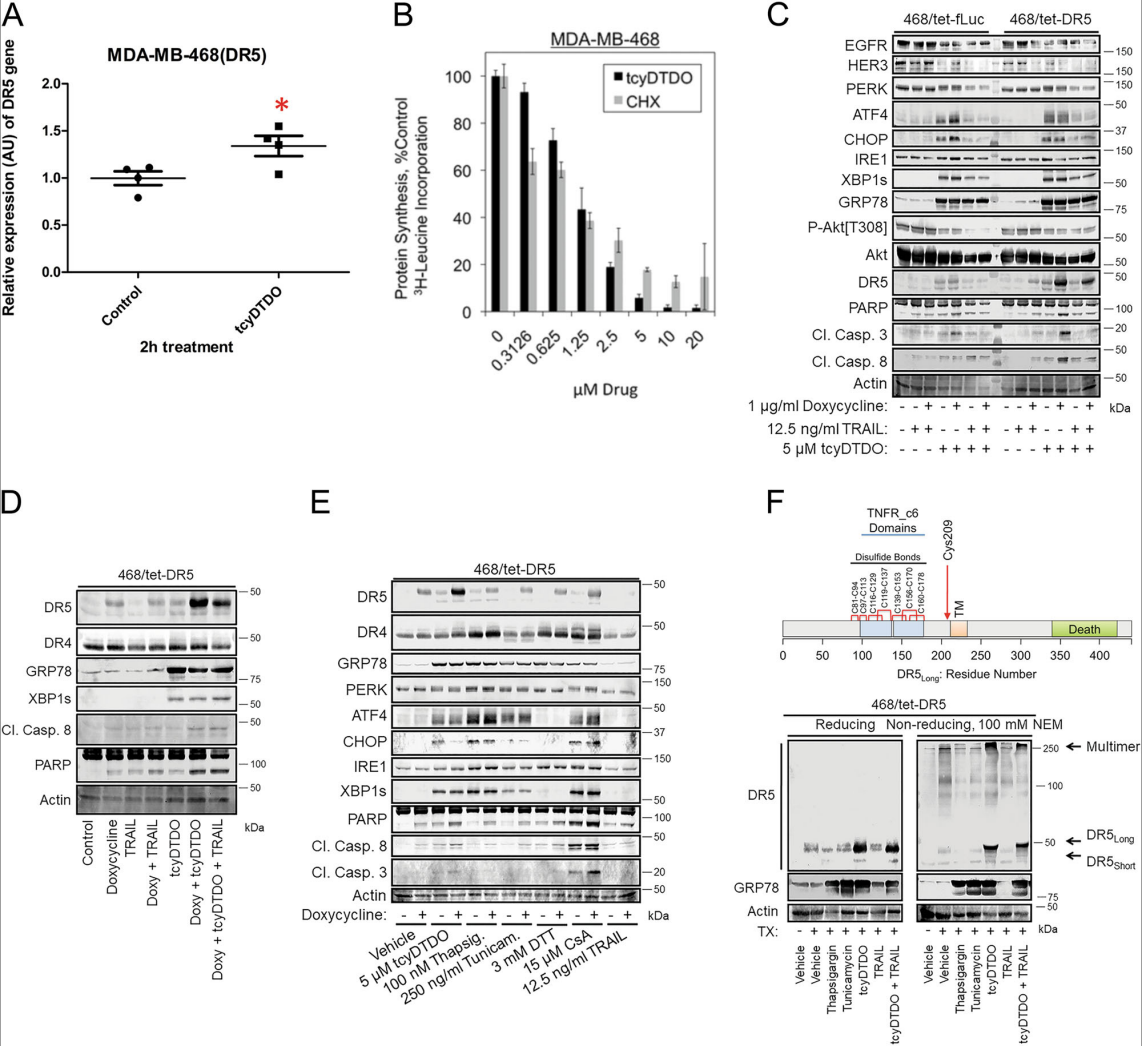
DDAs upregulate DR5 through transcriptional and post-transcriptional mechanisms. **a** MDA-MB-468 cells were treated with DMSO or 5 µM tcyDTDO for 2 h. Total RNA was isolated from each sample and converted to cDNA using reverse transcriptase. The mRNA levels of DR5 were measured using real-time qPCR, and relative mRNA expression was calculated. Fold change was calculated by normalizing all values to the untreated group. *T* test showed *p* = 0.0395. **b** Protein synthesis was assessed by ^3^H-leucine incorporation in MDA-MB-468 cells treated with the indicated concentrations of tcyDTDO or cycloheximide (CHX). **c** Immunoblot analysis of MDA-MB-468 cells engineered to express DR5 in a tetracycline-inducible manner (468/tet-DR5) and the corresponding control cell line (468/tet-fLuc) after the indicated 24-h treatments. **d** Immunoblot analysis of 468/tet-DR5 cells treated separately or with combinations of 1 µg/ml doxycycline, 12.5 ng/ml TRAIL, and 5 µM tcyDTDO for 24 h. **e** Immunoblot analysis of 468/tet-DR5 cells treated with the indicated agents for 24 h. **f** Upper panel. Diagram based on DR5 crystal structures showing the presence of seven intramolecular disulfide bonds and an unpaired cysteine residue in the extracellular domain of DR5. Lower panel. Immunoblot analysis of DR5 from 468/tet-DR5 cells treated for 24 h as indicated under nonreducing and reducing conditions. Cell extraction in the presence of *N*-ethylmaleimide NEM (100 mM) was used to limit thiol–disulfide exchange under nonreducing conditions.

ER stress increases the transcription of the gene encoding DR5 through activation of transcription factors ATF4 and CHOP^17–19^. Therefore, we examined whether tcyDTDO activates a previously described^13^ DR5 transcriptional reporter construct. TcyDTDO (5 µM) stimulated a several-fold increase in the activity of the DR5 reporter (Fig. S1A), suggesting a transcriptional mechanism. To validate this finding in cancer cells, time-course treatments (2–16 h) of MDA-MB-468 cells with 5 µM tcyDTDO were performed, and the mRNA level of DR5 was measured by RT-PCR and quantitative PCR. As shown in Figs. 2a and S1B, the increase in DR5 mRNA level was only detected at 2 h after tcyDTDO treatment, and only caused a 1.5-fold change. Since CHOP regulates DR5 expression, we constructed MDA-MB-468 and SKBR3 cell lines encoding either tetracycline-inducible control vector (468/tet-Puro) or CHOP (468/tet-CHOP), and knocked down CHOP in BT474 cells to study the effects of CHOP on DR5 levels in response to DDAs. CHOP overexpression failed to increase DR5 levels with or without DDA treatment (Fig. S1C, D), and CHOP knockdown only partly blocked DDA-mediated DR5 upregulation (Fig. S1E). These data suggest that tcyDTDO weakly increases the transcription of DR5. ER stress also reduces protein synthesis through PERK-dependent phosphorylation of eIF2α, so we examined the effect of tcyDTDO on protein synthesis. TcyDTDO suppressed protein synthesis in a concentration-dependent manner similarly to the protein synthesis inhibitor Cycloheximide (CHX) (Fig. 2b). Therefore, we hypothesized that DDAs regulate DR5 levels through post-transcriptional and post-translational mechanisms. We constructed stable MDA-MB-468 cell lines to inducibly express the long alternative splice variant of DR5 (468/tet-DR5), or firefly luciferase (468/tet-fLuc) as a control, to study the effects of DDAs on DR5 steady-state protein levels. In the 468/tet-DR5 cells, doxycycline + tcyDTDO robustly upregulated DR5 associated with strong induction of caspase 8 and 3 cleavage (Fig. 2c). In contrast, in the 468/tet-fLuc cells, doxycycline + tcyDTDO induced a weaker upregulation of DR5 and caspase 3 and 8 cleavage. Examining combinations of doxycycline, tcyDTDO, and TRAIL on 468/tet-DR5 cells demonstrated that the highest levels of the long form of DR5 were achieved by combining doxycycline and tcyDTDO (Fig. 2d), suggesting that tcyDTDO may stabilize DR5 at the protein level. These treatments did not alter the levels of DR4 (Fig. 2e). Comparison of tcyDTDO with other activators of the UPR demonstrated that at similar levels of ER stress induction, tcyDTDO produced the highest level of steady-state DR5 (Fig. 2e). Interestingly, levels of DR4 were increased by ER stressors that disrupt Ca^2+^ homeostasis (thapsigargin), reduce disulfide bonds (dithiothreitol (DTT)), and inhibit proline *cis-/trans*-isomerization (cyclosporine A (CsA)), while DR4 levels were not significantly altered by tcyDTDO. The DR5 extracellular domain contains seven disulfide bonds^20,21^ and DR5_Long_ contains an additional unpaired Cys, Cys 209, that is not present in DR5_Short_ (Fig. 2f, upper panel). Based on our previous proposal that DDA actions are mediated through effects on disulfide bond formation^4^, we examined if tcyDTDO alters the disulfide-bonding pattern of DR5. Immunoblot analysis of 468/tet-DR5 samples, which were prepared under reducing or nonreducing conditions in the presence of 100 mM *N*-ethylmaleimide (NEM) to prevent disulfide exchange after lysis, was used to compare the effects of multiple ER stress inducers on DR5 (Fig. 2f, lower panel). Analysis under reducing conditions revealed that thapsigargin, tunicamycin, and tcyDTDO induced comparable ER stress as measured by GRP78, but tcyDTDO had the strongest effect in upregulating DR5. Analysis under nonreducing conditions indicated that only tcyDTDO treatment shifted migration of DR5_Long_ monomer and increased accumulation of the disulfide-bonded multimeric form of DR5 at the top of the gel. MG132 and b-AP15 induce DR5 accumulation by inhibiting the proteasome and proteasome-associated deubiquitinases, respectively^13,22–24^. TcyDTDO, but not thapsigargin, tunicamycin, b-AP15, MG132, or TRAIL, reduced the electrophoretic ability of both DR5_Long_ and DR5_Short_ under nonreducing conditions in various cell lines (Fig. S2A–C). These results suggest that tcyDTDO altered the disulfide-bonding pattern of DR5_long_, and increased the formation of DR5 multimers by promoting the formation of intermolecular disulfide bonds.

### DDA upregulation of DR5 correlates with sensitization to TRAIL

**Fig. 3:**
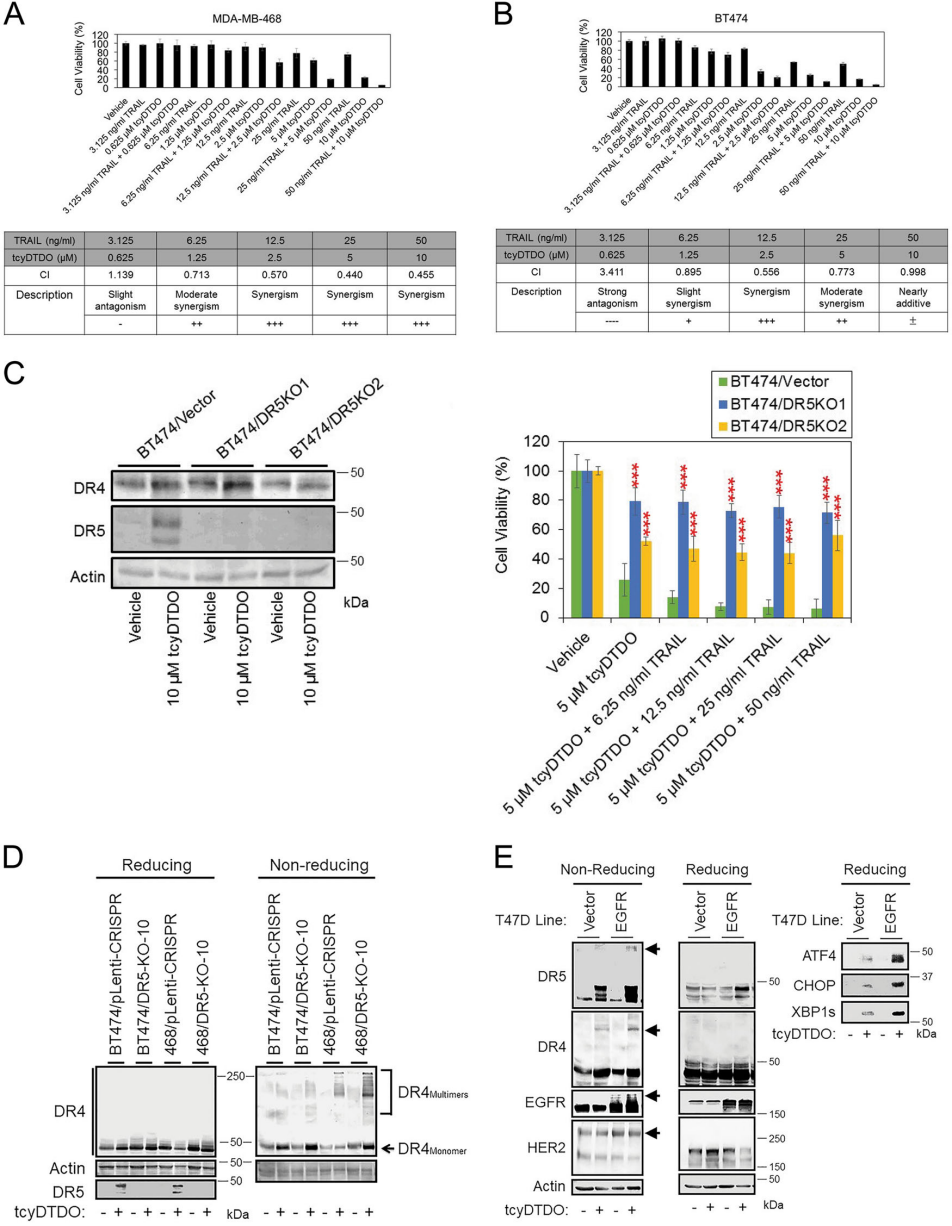
DDA upregulation of DR5 correlates with sensitization to TRAIL. **a**, **b** MTT cell viability assays performed on MDA-MB-468 and BT474 cells, respectively, after 72 h of the indicated treatments (upper panel) and analyzed using the Chou–Talalay method to calculate combination indices (CIs) (lower panel). [CIs: =1, >1, and <1 represent additivity, antagonism, and synergy, respectively.] Graphs represent the average of quadruplicate determinations ± standard deviation. **c** Left panel. Immunoblot analysis of BT474 control and DR5-knockout (DR5-KO) clones after a 24-h treatment with vehicle or 10 µM tcyDTDO. Right panel. MTT cell survival assays performed on BT474 control and DR5 -knockout clones after 72 h of the indicated treatments. **d** Immunoblot analysis showing comparison of DR4 oligomerization in MDA-MB-468 and BT474 vector control or DR5-knockout clones. **e** The indicated T47D cell lines were treated in the presence or absence of 5 µM tcyDTDO for 24 h. Samples were collected under reducing and nonreducing conditions for immunoblot analyses.

Since DDAs stabilize DR5 protein level through a post-transcriptional mechanism, we investigated whether DDA sensitizes cancer cells to TRAIL. Cell viability studies were performed in MDA-MB-468 (Fig. 3a) and BT474 (Fig. 3b) cells and synergy was analyzed using the Chou–Talalay method^25,26^. The results showed that the combination of tcyDTDO and TRAIL induces strong synergy in killing both cell lines. CRISPR/Cas9-mediated genome editing was used to generate DR5-knockout BT474 clones that were compared with control cells (Fig. 3c, left panel). Cell viability assays confirmed that ablation of DR5 expression reduced the sensitivity of cells to tcyDTDO treatment, or combined tcyDTDO/TRAIL treatment (Fig. 3c, right panel). Loss of DR5 expression did not fully rescue cell death from tcyDTDO + TRAIL treatments, so we hypothesized that DR4 might be activated by tcyDTDO after DR5 was knocked out. Vector control cells and DR5-knockout clones from different cell lines were treated in the presence or absence of tcyDTDO, and cell lysates were prepared in reducing or nonreducing conditions (Fig. 3d). TcyDTDO did not upregulate the total protein levels of DR4, but strongly induced DR4 oligomerization in DR5-knockout cells (Fig. 3d). TcyDTDO more strongly stimulated DR5 oligomerization in T47D/EGFR than in T47D/vector cells, suggesting that EGFR overexpression potentiates the effects of tcyDTDO on DR5 (Fig. 3e). Together, the results in Fig. 3 demonstrate that tcyDTDO sensitizes cancer cells to TRAIL through DR5 upregulation and oligomerization.

### DDAs kill Lapatinib-resistant breast cancer cells

**Fig. 4:**
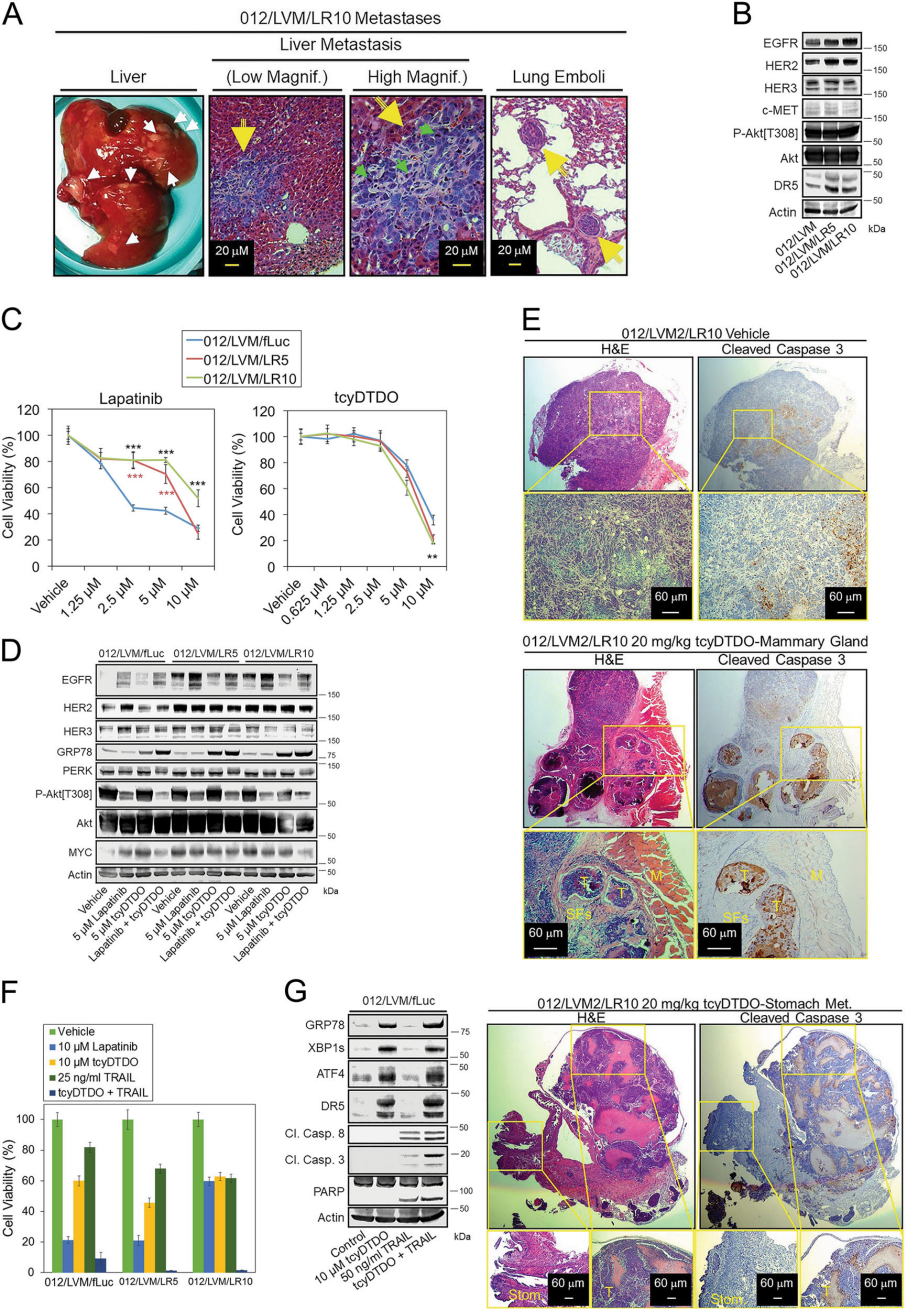
DDAs kill Lapatinib-resistant cancer cells. **a** Micrographs of liver metastases and lung tumor emboli generated in mice by a HCI-012 cell line selected for liver metastasis (LVM) and growth in the presence of 10 µM Lapatinib (LR10). **b** Immunoblot analysis of parental liver metastatic HCI-012 cells (012/LVM), or sublines selected for growth in 5 (LR5) or 10 µM (LR10) Lapatinib. **c** MTT cell viability assays on the indicated cell lines after treatment for 72 h with increasing concentrations of Lapatinib (left panel) or tcyDTDO (right panel). **d** Immunoblot analysis of the indicated cell lines treated for 24 h with the specified concentrations of Lapatinib, tcyDTDO, or Lapatinib + tcyDTDO. **e** Tumor-bearing mice were randomly separated into two groups of four mice each and treated for 5 days with either vehicle (DMSO) or 20 mg/kg of tcyDTDO. Mice were killed at day 5 (3 h after treatments) and tumor samples and metastatic lesions were collected for hematoxylin and eosin (H&E) staining and cleaved caspase 3 staining. Abbreviations used include M, muscle tissue; T, tumor tissue; SFs, stromal fibroblasts. **f** MTT cell viability assays performed after a 72-h treatment with 10 µM Lapatinib, 10 µM tcyDTDO, 25 ng/ml TRAIL, or tcyDTDO + TRAIL. **g** Immunoblot analysis of 012/LVM/fLuc cells treated for 24 h as specified. 012/LVM/fLuc cells were labeled using a lentiviral firefly luciferase vector as described in the “Materials and methods” section.

We previously isolated a cell line termed HCI-012^16^ obtained from a patient-derived xenograft (PDX) model of the same name. The HCI-012 xenograft originated from a patient with HER2+ breast cancer that metastasized and resisted treatment with cytotoxic and targeted (Lapatinib/Trastuzumab) therapy^27^. We selected sublines of the HCI-012 cells to model drug-resistant and metastatic breast cancers in two steps. We first isolated HCI-012 sublines capable of colonizing mouse lungs and liver after injection into either the tail vein or mammary gland (Fig. 4a). Second, continuous growth of the HCI-012 cells from liver metastases (012/LVM) in the presence of either 5 or 10 µM Lapatinib produced the Lapatinib-resistant sublines 012/LVM/LR5 and 012/LVM/LR10, respectively. Immunoblot analysis showed that the Lapatinib-resistant lines expressed higher levels of EGFR, HER2, and DR5 (Fig. 4b). Lapatinib cytotoxicity was reduced in the Lapatinib-resistant lines (Fig. 4c), but the sensitivity of all three cell lines to tcyDTDO was similar. Treatment of the control cell lines with Lapatinib increased expression of EGFR and HER2, and this effect was more dramatic in the Lapatinib-resistant lines (Fig. 4d). This suggests that the Lapatinib resistance may be due to Lapatinib upregulating EGFR and HER2 in addition to higher basal EGFR and HER2 levels in these cells. TcyDTDO partially decreased EGFR expression and Akt phosphorylation in control and Lapatinib-resistant cells. To study anticancer activities of tcyDTDO against Lapatinib-resistant tumors in vivo, eight mice were injected with HCI-012/LVM/LR10 cells in their mammary fat pads. After volumes of the primary tumors reached 100 mm^3^, mice were randomly assigned into two groups and treated once daily with vehicle or 20 mg/kg of tcyDTDO for 5 consecutive days by intraperitoneal injection. H&E staining of tumor tissue showed that both the primary and metastatic tumors from tcyDTDO-treated mice were necrotic, and large numbers of dying cells were observed surrounding the necrotic areas (Fig. 4e). Cleaved caspase 3 staining of serial sections confirmed that the apoptotic pathway was activated, especially surrounding the tumor necrotic areas (Fig. 4e). Importantly, tcyDTDO only induced apoptosis of cancer cells, while the normal muscle and stomach tissues remained intact, indicating selectivity of tcyDTDO toward cancer cells (Fig. 4e).

Combining tcyDTDO and TRAIL may be an effective strategy against Lapatinib-resistant 012/LVM lines, as the viability of these cells was somewhat decreased by treatment with either tcyDTDO or TRAIL, but tcyDTDO + TRAIL dramatically reduced the viability of all three lines (Fig. 4f). TcyDTDO and TRAIL-induced cell death was apparent as demonstrated by reduced cell numbers and apoptotic cell morphology in photomicrographs (Fig. S2D). Consistent with these observations, co-treatment with tcyDTDO and TRAIL resulted in maximal caspase 8, caspase 3, and PARP cleavage, indicative of the induction of apoptosis through the extrinsic death pathway (Fig. 4g).

**Fig. 5:**
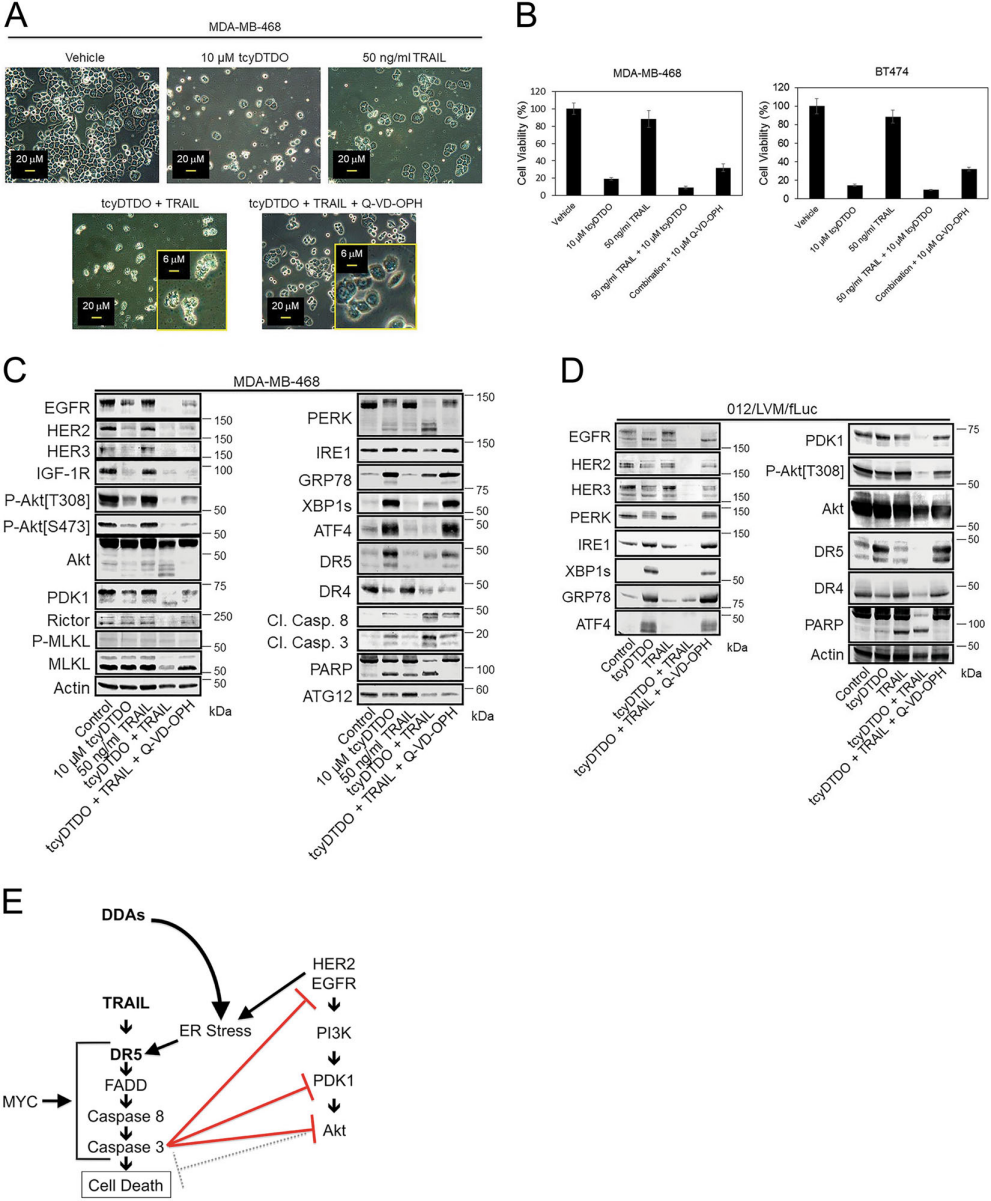
Caspases mediate DDA/TRAIL-induced downregulation of EGFR and PDK1. **a** Micrographs of MDA-MB-468 cells treated as indicated for 24 h showing cytotoxicity of tcyDTDO and TRAIL and prevention of cell death by the caspase inhibitor Q-VD-OPH (10 µM). **b** MTT assays were performed on MDA-MB-468 and BT474 cells with indicated treatments for 72 h. **c** Immunoblot analysis of MDA-MB-468 cells treated as in **a**. **d** Immunoblot analysis of 012/LVM/fLuc cells treated for 24 h as indicated. **e** Model for how DDAs and TRAIL reduce the levels of PERK, EGFR, PDK1, and Akt by inducing their caspase-dependent degradation.

While our data show that DDAs trigger extrinsic apoptosis, as indicated by the activation of DR5, caspases 8 and 3, and PARP cleavage, we do not always observe a direct correlation between increased activity of caspases 8 and 3 and DR4 or DR5 levels. There are several possible explanations for these discrepancies. Based on previous work^28^, DDA-mediated ER stress might activate caspase 12, which in turn could activate caspase 3. It is also possible that DDA-mediated ER stress increases the expression of inhibitor of apoptosis proteins (IAPs) such as cIAP1 or cIAP2^29^, which could impact the levels of activated caspases 8 and 3. Further, DR5 is required for maximal DDA induction of apoptosis (Figs. 1d and 3c), but death receptors can activate caspases 8 and 10^30^, so the relative DDA activation of caspases 8 and 10 could explain the absence of a perfect correlation between caspases 8 and 3 activation. However, a central role for caspase activation in mediating the anticancer actions of DDAs is indicated by the observations that (1) the caspase inhibitor Q-VD-OPH reverses many of the biochemical and biological responses to DDAs, with the exception of the DDA-mediated ER stress response (Fig. 5c), and (2) mouse tumor studies demonstrate strong DDA induction of caspase 3 cleavage by DDAs in cancer cells in vivo, but not in the surrounding normal tissues (Fig. 4e).

### Caspases mediate DDA/TRAIL-induced downregulation of EGFR and PDK1

As with the HCI-012 cell lines, tcyDTDO and TRAIL co-treatment killed MDA-MB-468 and BT474 cells more effectively than either agent alone (Fig. 5a and S2E). The caspase inhibitor Q-VD-OPH partially rescued the cells from tcyDTDO + TRAIL-mediated cell death (Fig. 5a, b). Rescue was associated with reversal of the rounded and apoptotic morphology of cells treated with tcyDTDO to the attached, spread morphology observed with the vehicle-treated cells. Immunoblot analysis indicated that tcyDTDO increased expression of DR5, but not DR4, and tcyDTDO + TRAIL strongly decreased EGFR, HER2, HER3, and IGF-1R expression and Akt phosphorylation (Fig. 5c). TcyDTDO + TRAIL induced the degradation of Akt, PDK1, and PERK based on the loss of the parent bands and formation of more rapidly migrating species. These effects were reversed by caspase inhibition. EGFR^31^ and Akt^32^ are caspase substrates. We are not aware of reports showing that PDK1 or PERK are degraded via a caspase-dependent mechanism. TRAIL + tcyDTDO produced near-complete PARP cleavage, which was reversed by Q-VD-OPH. In some systems, ER stress upregulates ATG12 and initiates autophagy^33^, or induces necroptosis^34^. In MDA-MB-468 cells, tcyDTDO + TRAIL did not increase expression of the autophagy marker ATG12 or the necroptosis marker phospho-MLKL, suggesting that cell death is predominantly through caspase-dependent apoptosis (Fig. 5c). Similarly, in 012/LVM/fLuc cells, tcyDTDO + TRAIL strongly decreased EGFR, HER2, HER3, and PDK1 levels and Akt phosphorylation, and increased PARP cleavage (Fig. 5d). These results indicate that DDAs and TRAIL cooperate to downregulate EGFR, HER3, PERK, Akt, and PDK1. Further, this downregulation correlates with the greatest degree of PARP cleavage, and these effects were reversed by caspase inhibition. In contrast, the ER stress and DR5 upregulation caused by tcyDTDO were not altered by caspase inhibition. The inability of Q-VD-OPH to overcome the ER stress response and the associated inhibition of protein synthesis and cell proliferation may explain its inability to restore cell viability to control levels. Together, the results suggest that the model in Fig. 5e operates in breast cancer cell lines, where stimulation of the TRAIL/DR5 pathway activates caspases that execute cell death in part through decreased expression of elements of the HER/PI3K/PDK1/Akt survival pathway.

### DDAs selectively kill oncogene-transformed cells

**Fig. 6:**
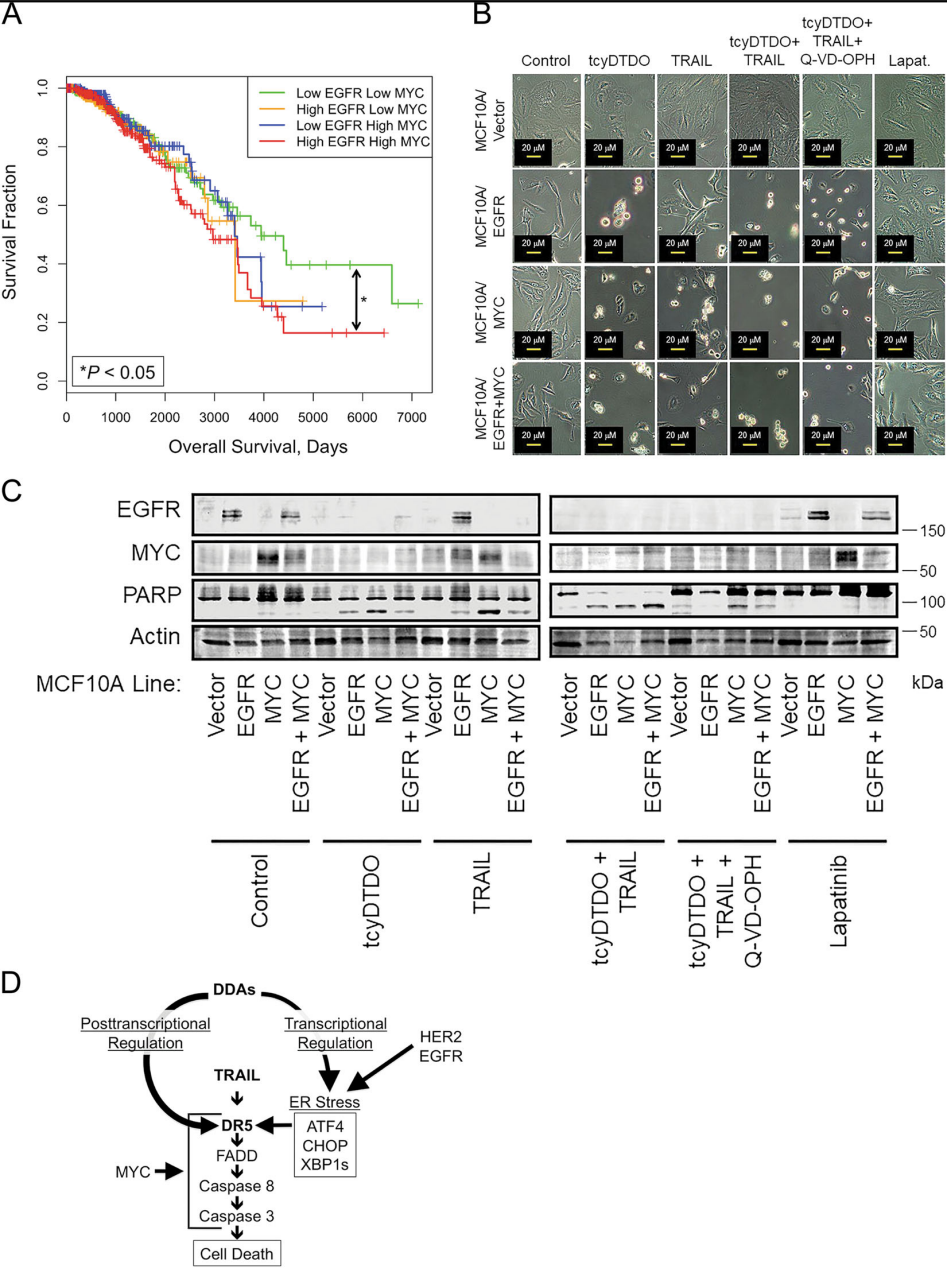
MYC or EGFR overexpression is sufficient to confer DDA cytotoxicity. **a** Kaplan–Meier plot showing that patients with tumors overexpressing both EGFR and MYC are associated with a significantly worse survival than are patients with tumors expressing low levels of both EGFR and MYC. Patients ranked based on the expression of EGFR and MYC were classified into four groups, named “low EGFR low MYC (*N* = 359)”, “low EGFR high MYC (*N* = 220)”, “high EGFR low MYC (*N* = 225)”, and “high EGFR high MYC (*N* = 372)”. Overall survival (OS) was compared among these groups. The *F* test was used to compare the variance between groups (*P* > 0.05, ns; *P ≤ 0.05, **P ≤ 0.01, ***P ≤ 0.001). **b** Micrographs showing the morphology of the indicated MCF10A stable cell lines after 24 h of treatment with 5 µM tcyDTDO, 12.5 ng/ml TRAIL, tcyDTDO + TRAIL, tcyDTDO + TRAIL + 10 µM Q-VD-OPH, or 10 µM Lapatinib. **c** Immunoblot analysis of stable MCF10A cell lines treated for 24 h as in **b**. **d** Model for how DDAs activate TRAIL/DR5-induced cell death in an oncogene-dependent manner. In the context of EGFR or HER2 overexpression, DDAs elevate ER stress resulting in transcriptional upregulation of DR5. Through a second mechanism, DDAs alter DR5 disulfide bonding to promote DR5 protein stabilization, oligomerization, and activation of pro-apoptotic signaling. Cytotoxicity of DDAs and TRAIL is also potentiated in MYC-overexpressing cells.

Cancer cells that overexpress EGFR or HER2 are particularly sensitive to DDAs^2,4^. The cancer lines chosen as representative of EGFR and HER2-overexpression, MDA-MB-468 and BT474, respectively, also exhibit MYC amplification^35–37^. Breast cancers with MYC and HER2 co-amplification are metastatic and exhibit a tumor initiating cell/cancer stem cell phenotype and poor prognosis^38^. Kaplan–Meier analyses demonstrate that breast tumors with high expression of both EGFR and MYC are likewise associated with decreased patient survival (Fig. 6a). To investigate whether overexpression of MYC increases the sensitivity of cancer cells to DDAs, we engineered MCF10A human mammary epithelial cells to overexpress EGFR, MYC, or both proteins using retroviral vectors. Photomicrographs and immunoblot analysis indicate that neither tcyDTDO nor TRAIL induced changes in cell morphology or PARP cleavage in the vector control cell line (Fig. 6b, c). In contrast, overexpression of EGFR or MYC either separately or together sensitized MCF10A cells to tcyDTDO, TRAIL, or tcyDTDO + TRAIL-induced cell death, but did not sensitize these cells to Lapatinib. The pan-caspase inhibitor Q-VD-OPH reversed the cell rounding and apoptotic morphology caused by tcyDTDO and tcyDTDO + TRAIL, but did not reverse tcyDTDO-mediated ER stress. Together, the results in Fig. 6 indicate that overexpression of either EGFR or MYC is sufficient to sensitize cells to tcyDTDO or TRAIL-mediated cell death. This is consistent with DDAs and TRAIL selectively killing oncogene-transformed cells through overlapping mechanisms, and suggest that MYC/EGFR may serve as predictive biomarkers of tcyDTDO efficacy.

## Discussion

Great effort has been expended in testing soluble TRAIL and DR5 agonist antibodies as anticancer agents (reviewed in refs. ^36,37^). These trials have not demonstrated dramatic anticancer activity, due to short in vivo half-lives and trimer dissociation for soluble TRAIL agonists^38–40^, and the inability of bivalent agonist antibodies to trigger the same receptor trimerization induced by soluble TRAIL. DR4 and DR5 can be inactive in cancer cells due to decreased expression of the proteins^41^, constitutive protein internalization^42–44^, or alterations in post-translational modifications^45–47^. Potential solutions include generation of more stable trimeric forms of TRAIL^48–51^, and the induction of increased DR5 expression through pharmacological agents that induce ER stress to activate *DR5* transcription^6–9,52,53^. Other strategies have included increasing DR5 half-life by decreasing its proteasomal degradation by inhibiting the proteasome^23,54,55^ or proteasome-associated deubiquitinases (DUBs)^24^. We are not aware of pharmacological approaches that: (a) cause DR5 accumulation and oligomerization, and (b) stimulate downstream caspase activation and cancer cell death through mechanisms involving altered DR5 disulfide bonding.

Our results suggest the model in Fig. 6d where DDAs activate TRAIL/DR5 signaling through two mechanisms. First, DDAs induce ER stress that is strongly potentiated by EGFR or HER2 overexpression (Fig. 1C and ref. ^2^), resulting in induction of the UPR and increased DR5 expression. Previous reports have shown transcriptional upregulation of DR5 by various ER stressors^6–9,52,53^. TcyDTDO or RBF3^4^ upregulation of DR5 is not blocked by a PERK kinase inhibitor (GSK2606414^56^), even though upregulation of ATF4 and CHOP is blocked (Fig. S3A). PERK inhibition does not affect tcyDTDO upregulation of GRP78 or XBP1s (Fig. S3B), so XBP1s or ATF6 may participate in DR5 upregulation in response to tcyDTDO.

Second, DDAs act distinctly from other ER stress inducers to stabilize steady-state DR5 protein levels and induce DR5 multimerization. These mechanisms may explain the ability of tcyDTDO to induce cleavage of caspases 8, 3, and PARP in the absence of TRAIL, and to potentiate the cytotoxicity of TRAIL. This is the first evidence that altering DR5 disulfide bonding favors multimerization and increased downstream signaling. A recent report showed that deletion of the extracellular domain of DR5 permits oligomerization mediated by the transmembrane domain^57^. Thus, the extracellular domain prevents receptor oligomerization and downstream signaling in the absence of TRAIL. The extracellular domains of DR5 and DR4 contain seven disulfide bonds (see Fig. 2f) that mediate their proper folding. We speculate that DDAs alter the patterns of DR5 and DR4 disulfide bonding to allow their oligomerization and downstream signaling in the absence of TRAIL.

DDAs are selective against cancer cells over normal cells in vitro and in vivo (herein (Fig. 6c) and elsewhere^2,4^). Multiple mechanisms explain the oncotoxicity of DDAs. First, DDAs selectively induce ER stress, with associated DR5 upregulation, in the context of EGFR or HER2 overexpression (Fig. 1c). Second, breast cancer cells often overexpress MYC, which strongly enhances apoptosis through the TRAIL/DR5 pathway^58–61^. Third, TRAIL kills cancer cells without affecting nontransformed cells^11,12,35,62^. Interestingly, HCI-012 lines selected for Lapatinib resistance exhibit high basal EGFR and HER2 expression, and Lapatinib treatment of these lines further elevates EGFR and HER2 levels. In addition, the resistant lines show higher MYC levels. This may explain why resistance to Lapatinib is not associated with DDA resistance.

## Materials and methods

### Cell culture, preparation of cell extracts, and immunoblot analysis

The cell lines MCF10A, MDA-MB-468, BT474, T47D, SW480, and DU145 were purchased from American Type Culture Collection (ATCC) (Manassas, VA). The HCI-012 cell line was derived from a HER2+ patient-derived xenograft that was originally isolated from a patient as detailed previously^2,27^. MCF10A cells were cultured as described previously^63^. Unless otherwise indicated, cancer cell lines were grown in Dulbecco's modified Eagle’s medium (GE Healthcare Life Sciences, Logan, UT) supplemented with 10% fetal bovine serum (10% FBS–DMEM) in a humidified 37 °C incubator with 5% CO_2_. Cell lysates were prepared as described previously^64^. Immunoblot analysis was performed by employing the following antibodies purchased from Cell Signaling Technology (Beverly, MA) [Akt, #4691; P-Akt[T308], #13038; P-Akt[S473], #9271; ATF4, #11815; EGFR, #4267; HER2, #2165; HER3, #4754; IRE1, #3294; XBP1s, #12782; PARP, #9532; PERK, #5683; GRP78, #3177; CHOP, #2895; DR5, #8074; DR4, #42533; PDK1, #5662; Cleaved Caspase 8, #9496; Cleaved Caspase 3, #9664; MET, #3127; PERK, #9101, Rictor, #2140; MLKL, #14993; P-MLKL, #91689; PDI, #3501] and Santa Cruz Biotechnology (Santa Cruz, CA) [IGF-1R, sc-713; MYC, sc-764; ERK, sc-93; Actin, sc-1616-R]. P-IRE1[Ser724] (nb100-2323ss) antibody was from Novus Biologicals.

The following reagents were purchased from the indicated sources: Tunicamycin, 2-Deoxyglucose: Sigma-Aldrich (St. Louis, MO); 2-Aminoethoxydiphenyl borate (2-APB): StressMarq Biosciences (Cadboro Bay, Victoria, Canada); Thapsigargin: AdipoGen (San Diego, CA); Cycloheximide, Rapamycin: EMD Biosciences (Darmstadt, Germany); Lapatinib: Selleck Chemicals (Houston, TX); Doxycycline: Enzo Life Science (Farmingdale, NY); CCF642, TORIN1, and dithiothreitol (DTT): TOCRIS Bioscience (Minneapolis, MN); Cyclosporin A (CsA): Bioryt (Atlanta, GA); LOC14, PERK Inhibitor I (GSK2606414): Calbiochem (Burlington, Massachusetts); Q-VD-OPH, Kifunensine: Cayman Chemical (Ann Arbor, MI); *N*-ethylmaleimide (NEM): Thermo Fisher Scientific (Grand Island, NY); MG132: InvivoGen (San Diego, CA); b-AP15: MedKoo Biosciences (Chapel Hill, NC); TRAIL: PEPROTECH (Rocky Hill, NJ).

### Construction of stable cell lines using recombinant retroviruses and lentiviral shRNAs

Stable cell lines were constructed as follows: retroviral vectors encoding EGFR (Plasmid 11011^65^) and HER2 (Plasmid 40978^66^) were purchased from Addgene (Cambridge, MA). Stable MCF10A cell lines ectopically expressing EGFR, MYC, or HER2 were prepared according to the methods used in a previous report^64^. MYC Plasmid 17758 was obtained from Addgene^67^ to allow coexpression of EGFR and MYC using puromycin and zeocin selection, respectively.

T47D/Vector, T47D/EGFR, T47D/HER2, and T47D/EGFR/HER2 cell line construction was described in our previous report^3,68^. The HCI-012/LVM cells were isolated from mouse liver metastases using the conditional cell reprogramming approach of Schlegel and colleagues^69^ as detailed in a previous report^2^. Lapatinib-resistant sublines 012/LVM/LR5 and 012/LVM/LR10 were developed by growing HCI-012/LVM cells in the continuous presence of either 5 or 10 µM Lapatinib. Lentiviral DR5 shRNA constructs were from the TRC Lentiviral shRNA Libraries from Thermo Scientific. Labeling cells with firefly luciferase (fLuc) was performed using Addgene Plasmid 47553^70^. The protocol employed for developing stable cell lines with lentiviral shRNAs was from the Thermo Scientific website: https://www.thermofisher.com/us/en/home/references/gibco-cell-culture-basics/transfection-basics.

### CRISPR/Cas9-mediated genome editing

Four guide RNA sequences were designed to target the human DR5 gene (GenBank accession number: AF012628) using the online CRISPR Design tool at http://crispr.mit.edu. Sequences of the four DR5 oligonucleotide pairs are as follows: 5′-CACCGAGAACGCCCCGGCCGCTTCG-3′ and 5′-AAACCGAAGCGGCCGGGGCGTTCT-3′; 5′-CACCGCCTTGTGCTCGTTGTCGCCG-3′ and 5′-AAACCGGCGACAACGAGCACAAGGC-3′; 5′-CACCGCGCGGCGACAACGAGCACAA-3′ and 5′-AAACTTGTGCTCGTTGTCGCCGCGC-3′; 5′-CACCGTTCCGGGCCCCCGAAGCGGC-3′ and 5′-AAACGCCGCTTCGGGGGCCCGGAAC-3′. Oligonucleotide pairs were annealed, phosphorylated with polynucleotide kinase, and cloned into the BsmBI site of LentiCRISPRv2 (Addgene #52961). Cloning of gRNA sequences into LentiCRISPRv2 was verified by sequence analysis. Viruses expressing the four different DR5- directed gRNAs were packaged using HEK 293T cells. To produce stable cell lines, target cell lines were subsequently infected with lentivirus and selected with 5 μg/ml puromycin, as described previously^64,68^. Clonal-knockout cell lines were isolated by limiting dilution and characterized by immunoblot analysis.

### RT-PCR and RT-qPCR

Total RNA was extracted with Trizol Reagent (Invitrogen #15596-018) according to the manufacturer’s protocol. Total cellular RNA was used to synthesize first-strand cDNA by using the PCR conditions listed: 25 °C for 10 min, 42 °C for 30 min, and 95 °C for 5 min. PCR was subsequently performed using either DR5 or β-actin primers. The primer sequences for DR5 are 5′- TCCACCTGGACACCATATCTCAGAA-3′ and 5′-TCCACTTCACCTGAATCACACCTG-3′ and the primer sequences for β-actin are 5′-GGATGCAGAAGGAGATCAC-3′ and 5′-AAGGTGGACAGCGAGGCCAG-3′. The PCR products were visualized on 3% agarose gels with ethidium bromide staining under UV transillumination with a digital camera system, and quantified using NIH ImageJ. Real-time qPCR was performed with the QuantStudio 6 Flex system (Thermo Fisher #4485691) using PowerUp from Invitrogen (https://www.thermofisher.com/order/catalog/product/A25742). The expression level of DR5 was normalized to β-actin, and the cycle threshold value of the sample was used to calculate the relative gene expression level = 2^−(Ct target−Ct actin)^. The relative change in gene expression compared with the control group was expressed as fold change, and calculated by the 2^−ΔΔCt^ method^71^.

### Protein synthesis assays

Protein synthesis assays were carried out as detailed previously^72^ using ^3^H-Leucine (cat. # NET460001MC) obtained from Perkin Elmer (Waltham, MA).

### Luciferase transcriptional reporter assays

Reporter assays were performed as described previously^2^ using the DR5 reporter construct obtained from Addgene (Plasmid 16012^73^).

### Cell viability assays

Cell viability was evaluated using the MTT (3-(4,5-dimethylthiazol-2-yl)-2,5-diphenyltetrazolium bromide) assay. MTT assays were carried out according to the manufacturer’s instructions (kit CGD1, Sigma-Aldrich, St. Louis, MO).

### In vivo tumor studies, histochemical, and immunohistochemical analysis

Mice were housed, maintained, and treated in the Animal Care Service Center at the University of Florida in accordance with the Institutional Animal Care and Use Committee (protocol number: 201608029). NOD-SCID-gamma (NSG) mice were obtained from Jackson Laboratories (Bar Harbor, ME). Breast cancer liver metastasis was initiated by injecting 1 × 10^6^ cancer cells into the #4 mammary fat pads, or 200,000 cells into the lateral tail veins of adult female NSG mice.

Tissue samples were fixed in 4% paraformaldehyde/PBS and paraffin embedded. Tissue sectioning, hematoxylin and eosin (H&E) staining, and immunohistochemical staining for 6 cleaved Caspase 3 (Cat. #9664, Cell Signaling Technology) were performed by the University of Florida Molecular Pathology Core (https://molecular.pathology.ufl.edu/).

### Statistics

In Fig. 6a data obtained from The Cancer Genome Atlas (TCGA; https://tcga.xenahubs.net/download/TCGA.BRCA.sampleMap/HiSeqV2_PANCAN.gz) [dataset ID: TCGA_BRCA_exp_HiSeqV2_PANCAN] were used to examine the relationships between tumor expression of EGFR and MYC at the mRNA level and patient survival. Gene expression data for 1176 patients with invasive breast carcinoma was measured by RNAseq and mean-normalized across all TCGA cohorts. EGFR and MYC were the two genes of interest in our study. Patients ranked based on the expression of EGFR and MYC were classified into four groups, named “low EGFR low MYC (*N* = 359)”, “low EGFR high MYC (*N* = 220)”, “high EGFR low MYC (*N* = 225)”, and “high EGFR high MYC (*N* = 372)”. Overall survival (OS) was compared among these groups. The *F* test was used to compare the variance between groups (*P* > 0.05, ns; *P ≤ 0.05, **P ≤ 0.01, ***P ≤ 0.001).

Statistical analyses for synergies between drugs were performed using CalcuSyn software (http://www.biosoft.com/w/calcusyn.htm). The combination index (CI) was calculated by applying the Chou–Talalay method and was used for synergy quantification^74^. Student’s *t* test was used for comparisons in both in vitro and in vivo experiments. All *P* values are two-tailed, and both *P* values and statistical tests are mentioned in either figures or legends.

### Chemical syntheses of DDAs

RBF3 and tcyDTDO were prepared as described previously^4,16^.

## Supplementary information

Supplemental Material
