# Supplementary material for: Disulfide bond-disrupting agents activate the tumor necrosis family-related apoptosis-inducing ligand/death receptor 5 pathway
Source: Cell Death Discov. 2019 Dec 10;5:153. doi: 10.1038/s41420-019-0228-9 (PMC6904486; doi:10.1038/s41420-019-0228-9)
Supplement: Supplementary file 1 — Supplemental Material [file 41420_2019_228_MOESM1_ESM.pdf]

Supplemental Material

Disulfide Bond Disrupting Agents Activate the Tumor Necrosis Family-Related Apoptosis-Inducing Ligand/Death Receptor 5 Pathway

[1]Mengxiong Wang, [1]Mary E. Law, [1]Bradley J. Davis, [2]Elham Yaaghubi, [2]Amanda F. Ghilardi, [2]Renan B. Ferreira, [5]Chi-Wu Chiang, [1,4]Olga A. Guryanova, [1]Daniel Kopinke, [3,4]Coy D. Heldermon, [2,4]Ronald K. Castellano[#], and [1,4]Brian K. Law[#]

Departments of [1]Pharmacology, [2]Chemistry, and [3]Medicine, and the [4]UF-Health Cancer Center, University of Florida, Gainesville, FL, [5]Institute of Molecular Medicine, College of Medicine, and Center for Infectious Disease and Signaling Research, National Cheng Kung University, Tainan, Taiwan

[#]Co-Contributing Authors

Table of Contents

Supplemental figure (S) 1: DDAs increase DR5 mRNA .... **Page 2**

S2: TcyDTDO-induced DR5 electrophoretic mobility shifts .... **Page 3**

S3: TcyDTDO upregulation of DR5 is not blocked by PERK inhibition .... **Page 4**

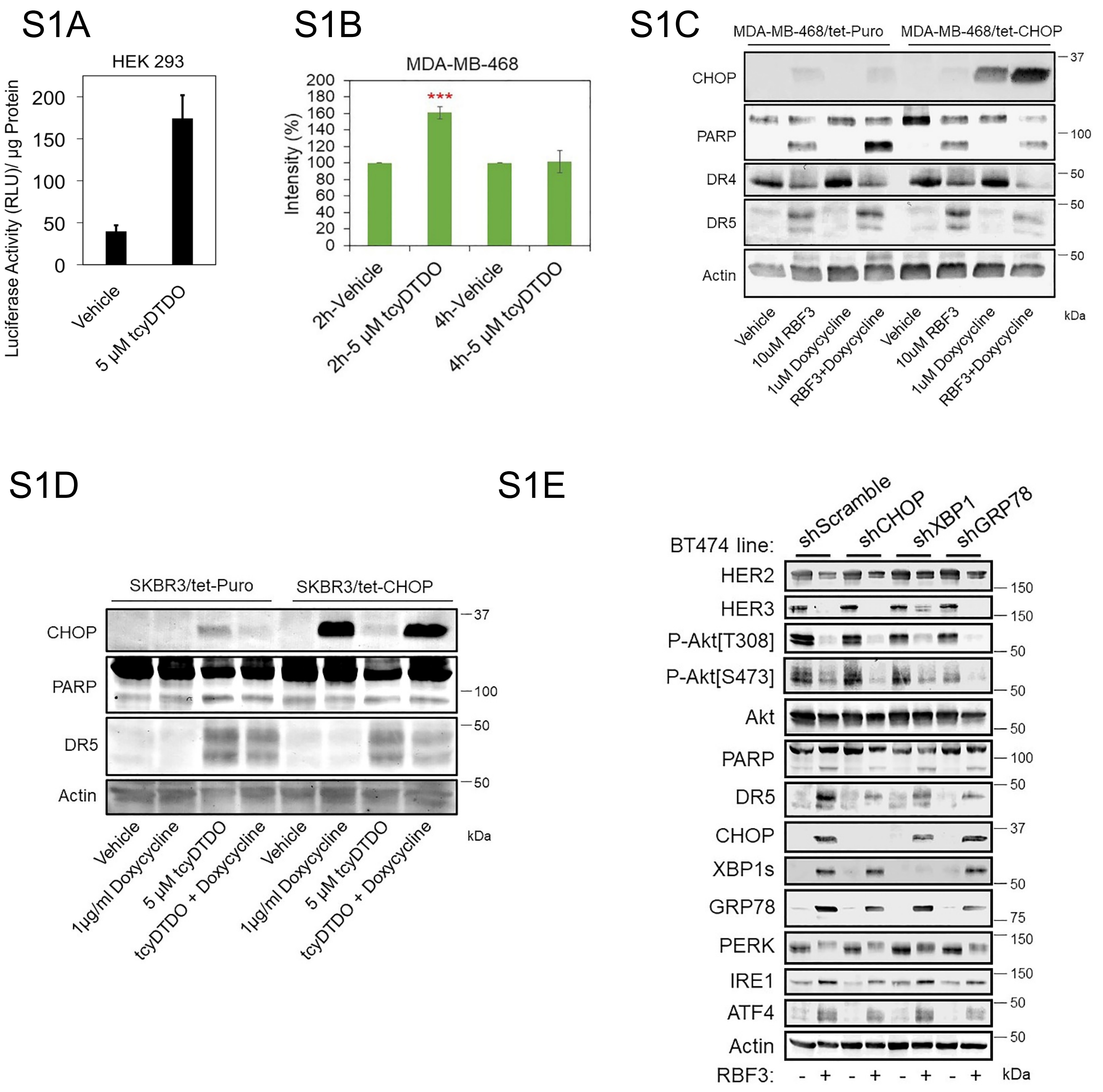

Fig. S1- DDAs increase DR5 mRNA. A. Luciferase transcriptional reporter assays were performed in HEK 293 cells using a DR5 transcriptional reporter construct. Cells were treated with DMSO vehicle or 5 μM tcyDTDO for 24 h prior to luciferase assays. B. RT-PCR of DR5 was performed on the MDA-MB-468 cell line with indicated treatments. DR5 mRNA levels were measured by NIH ImageJ analysis and were normalized to corresponding β-Actin mRNA levels. C., D. Immunoblot analysis of MDA-MB-468 and SKBR3 cells engineered to express CHOP in a tetracycline-inducible manner (468/tet-CHOP) and the corresponding control cell line (468/tet-Puro) after the indicated 24 h treatments. E. Immunoblot analysis of the indicated stable BT474 shRNA-mediated knockdown cell lines after 24 h treatment with vehicle or 20 μM RBF3. Error bars in S1A and S1B represent standard deviation.

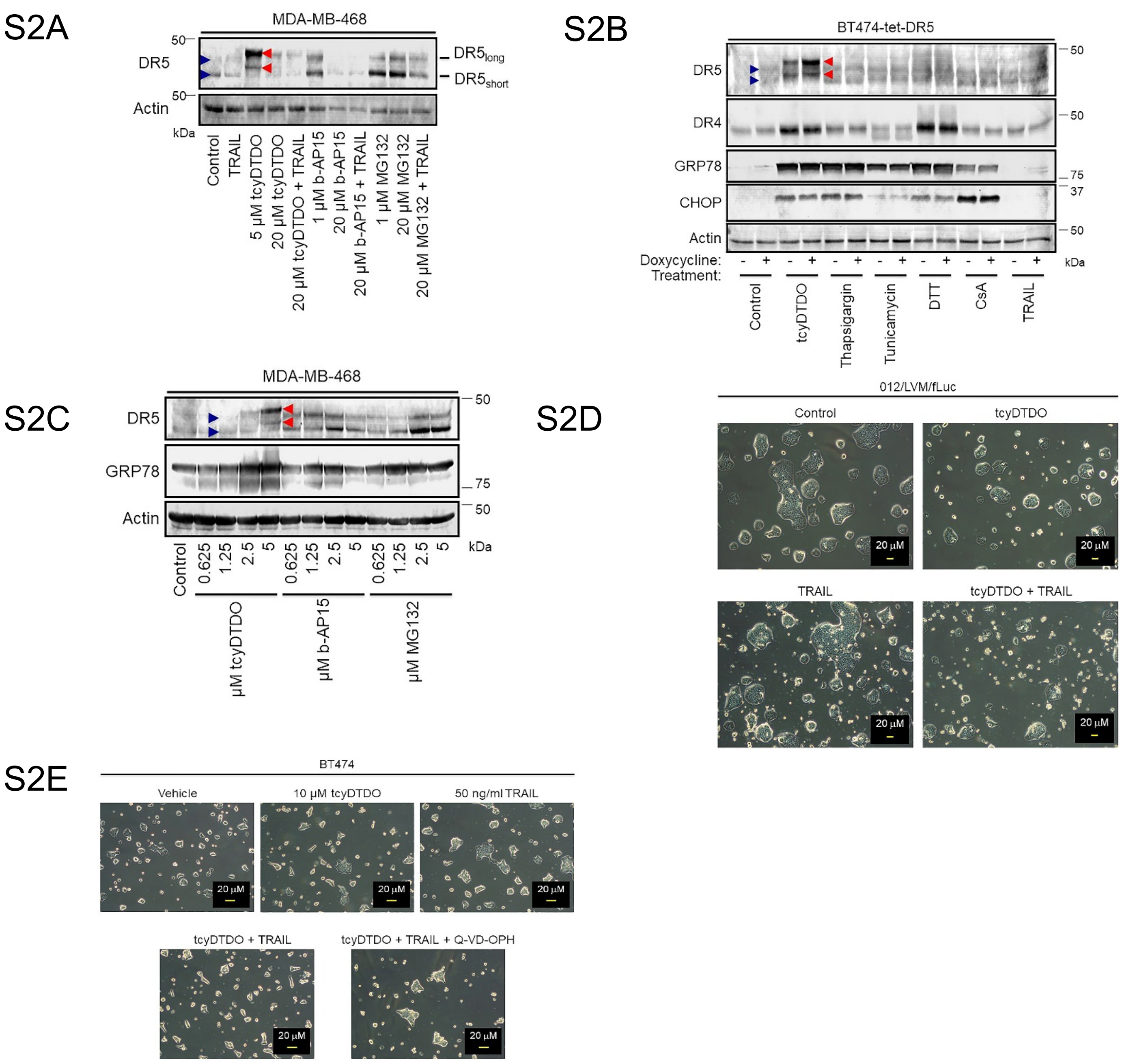

Fig. S2- TcyDTDO-induced DR5 electrophoretic mobility shifts. A. MDA-MB-468 cells were treated for 24 h with 12.5 ng/ml TRAIL and the indicated concentrations of the other agents, and extracted with boiling 2X SDS-PAGE sample buffer containing 100 mM NEM. Samples were then sonicated, clarified by centrifugation, and analyzed by immunoblot. B. BT474 cells engineered to express DR5 in a doxycycline-inducible manner (BT474-tet-DR5) were treated for 24 h with or without 1 µg/ml doxycycline in the presence or absence of 5 µM tcyDTDO, 100 nM Thapsigargin, 250 ng/ml Tunicamycin, 3 mM DTT, 15 µM CsA, or 12.5 ng/ml TRAIL as indicated. Cells were extracted and analyzed as described in Fig. S2A. C. MDA-MB-468 cells were treated for 24 h as indicated and analyzed by immunoblot as described in Fig. S2A. In A-C, blue arrows indicate bands corresponding to the long and short forms of DR5 under control conditions and red arrowheads show bands corresponding to DR5 migration after treatment with tcyDTDO. Note that tcyDTDO reduces the migration of both DR5 isoforms. D. Micrographs of 012/LVM/fLuc cells treated for 24 h with vehicle (DMSO), 10 µM tcyDTDO, 25 ng/ml TRAIL, or tcyDTDO + TRAIL. E. Micrographs showing the morphology of BT474 cells after 24 h of treatment as indicated.

S3A

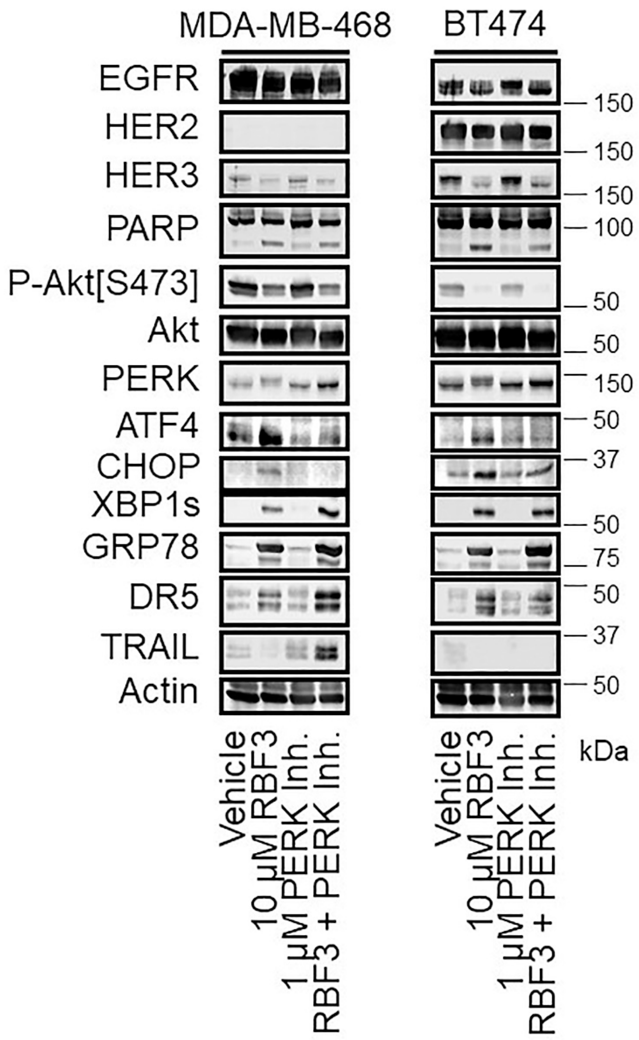

S3B

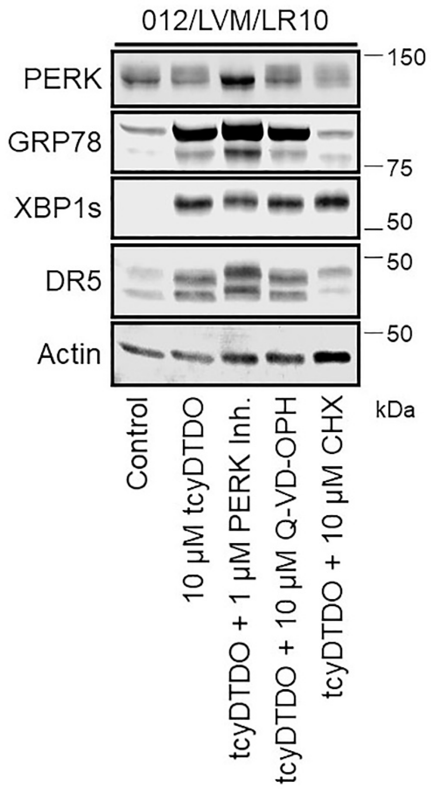

S3- TcyDTDO upregulation of DR5 is not blocked by PERK inhibition. A. MDA-MB-468 (left panel) or BT474 cells (right panel) were treated for 24 h as indicated and subjected to immunoblot analysis. B. 012/LVM/LR10 cells were treated for 24 h as indicated and subjected to immunoblot analysis.
